# Molecular Research on Vector-Borne Diseases of Medical Interest: From Bench to Application 2.0

**DOI:** 10.3390/ijms24097907

**Published:** 2023-04-26

**Authors:** Denis Sereno

**Affiliations:** Parasite Infectiology Research Group, Institut de Recherche Pour le Développement, Université de Montpellier, InterTryp, 34032 Montpellier, France; denis.sereno@ird.fr

Infectious diseases caused by parasites (malaria, leishmaniasis, trypanosomiasis, filariasis…), viruses (chikungunya, dengue, phlebovirus, etc.), or bacteria (bartonellosis, Lyme disease) are transmitted by a variety of arthropods belonging to the Culicidae, Simuliidae, Psychodidae, Ixodidae, Agarsidae, Pulicidae, Glossinidae, Reduviidae, Muscida, and Tabanidae families [1]. After decades, many vector-borne human illnesses have finally been controlled. However, climate change—which has resulted in the modification of weather patterns and an increase in extreme weather events—affects vector-borne disease outbreaks and is likely to have both short- and long-term effects on their transmission and infection patterns, affecting seasonal risk and broad geographic changes in disease occurrence [2,3]. Improving knowledge of the molecular basis of interactions between pathogens and their hosts (vertebrate and invertebrate) is necessary to enhance the detection of these pathogens and to foresee new diagnostic, therapeutic, or prophylactic alternatives.

The complexity of the subject in terms of the pathogen diversity, the transmission life cycle, and the host affected was hinted at in the first Special Issue of the *International Journal of Molecular Sciences* on “Molecular research on vector-borne diseases: from bench to application”. In this Special Issue, valuable publications on new biomarkers for the epidemiological survey of arthropod vector bites [4], as well as on the transcriptomic response to human complement protein in an arthropod vector [5] and during host viral infection [6], were provided for some vector-borne pathogens and diseases. Moreover, a paper on the potential of frog antimicrobial peptides to act against vector-borne pathogens [7] and a systematic review and meta-analysis on the possibility of non-invasive biopsy to be used for Trypanosomatidae pathogens detection were published [8]. Contributions included in the present update shed more light on many questions in this field; however, much work remains in attempting to translate the reported disclosures into the field.

This Special Issue of the *International Journal of Molecular Sciences*, entitled “Molecular Research on Vector-Borne Diseases of Medical Interest: From Bench to Application 2.0” includes a total of eight contributions—five original Articles and three reviews—providing new information about various aspects of vector-borne diseases, ranging from diagnostics to drug development. Extensive reviews on schistosomiasis in Southeast Asia, on fatty acid metabolism in *Leishmania* pathogens, and on isothermal DNA amplification as field diagnostic perspectives of Trypanosomatidae infection complete this Special Issue.

The first set of scientific articles and reviews focuses on new inhibitors with antiparasitic activity, further insight into *Leishmania* leishmaniolysin metalloproteinase diversity and activity, and a discussion and review of the unique fatty acid profile and metabolism of trypanosomatid parasites.

Histone deacetylase has emerged as a leader in treating cancer and various infectious diseases. A report by Jublot et al. [9] investigates a histone deacetylase (HDAC) inhibitor, previously synthesized [10,11] to act in vitro against a panel of *Toxoplasma* strains, and the liver and blood stages of *Plasmodium*, the causative agents of malaria, are investigated. The authors observed significant increases in mice survival during the acute phase of *T. gondii* infection, and demonstrated the efficiency of the HDAC inhibitor in controlling the formation of brain cysts in *T. gondii*-infected mice. The JF363 compound docking in the five annotated ME49 *T. gondii* HDAC-active sites displays a similar binding mode in the five paralogous structures and a similar micromolar range prediction of affinities. These results will support future development aimed at treating acute and chronic toxoplasmosis and developing anti-*Plasmodium* therapeutic interventions.

Leishmanolysin, or promastigote protease (PSP) or gp63, is an abundant parasite virulence factor surface glycoprotein of *Leishmania* promastigotes. The metalloprotease activity of gp63 is inhibited powerfully by metal chelators. Here, the three-dimensional structure of gp63 and its active site was modelized and used as a template to search for possible inhibitors in the literature and molecular databases. Molecular docking simulations of the generated protein–ligand complexes were computed. The individual energy of gp63 amino acids interacting with its ligands was quantified by ab initio calculations using Molecular Fractions with Conjugated Caps (MFCC). The MFCC analysis of each candidate allowed quantum balance calculations for protein interaction. A compound (L1), a 7-hydroxycoumarin derivative, obtains the best energy quantum balance result (−2 eV), followed by DETC, doxycycline, and 4-terpineol. In vitro experiments confirmed L1 antileishmanial activity against *L. amazonensis* promastigotes, low cytotoxicity against mammalian RAW and 3T3 cell lines, and a selectivity index of 149.19 and 380.64 µM, respectively. The L1 compound induced promastigote death by necrosis, with disruption in membrane integrity further documented by Spectroscopy Dispersion analysis. The L1 would be an effective compound against *L. amazonensis*, the etiologic agent of diffuse cutaneous leishmaniasis.

*Leishmania tarentolae*, a non-human pathogenic trypanosomatid, is frequently used as a heterologous protein expression system. Nevertheless, it can help delineate the underlying virulence and pathogenic mechanisms involved in human and animal leishmaniases. The repertoire of Leishmaniolysin genes in the genome of *L. tarentolae* was investigated by Ennes-Vidal et al. [12], and a total of 61 leishmanolysin sequences were retrieved. These are phylogenetically more related to the *L. major* leishmaniolysin than the *L. braziliensis* and *L. martiniquensis*. Surprisingly no proteolytic activity is detected. The 3D homology models based on the crystallographic structure of *L. major* ortholog were built with three *L. tarentolae* gp63 sequences. Molecular dynamics simulations disclosed a lower electrostatic negative potential than the *L. major* template for the three models constructed in silico. The *L. major* LmjF.10.0460 and the *L. tarentolae* LtaP10.0650 leishmanolysins genes were then cloned into the pLEXSY vector and transfected into *L. tarentolae* to allow their expression. The authors observed that *L. tarentolae* leishmanolysins harbor a weak enzymatic activity, three times less than *L. major* ones. This suggests that the lower electrostatic negative potential supported by *L. tarentolae* leishmanolysin contributes to the reduced proteolytic activity detected in this parasite species.

Characterizing enzymes allowing fatty (FA) acid synthesis by *Leishmania* opens perspectives on understanding their role in parasite biology and development. The review proposed by Leroux et al. [13] provides clues on FA composition profiles and the metabolism of the major lipids and phospholipids classes in *Leishmania* species responsible for cutaneous or visceral diseases. In *L. major*, *T. cruzi*, and *T. brucei*, in human and animal trypanosomatid pathogens, several enzymes involved in de novo FA synthesis have emerged as potential drug targets. Drug resistance to first-line antileishmanial compounds is documented [14,15,16,17]; but their implication in therapeutic failure is debated [18]. Changes in FA compositions are observed in several *Leishmania* species upon exposure to antileishmanial drugs and resistant *Leishmania* isolates. These changes are proposed as putative mechanisms for drug toxicity or drug resistance. The conversion of polyunsaturated fatty acids and their metabolites into inflammatory mediators and oxygenated metabolites, acting on metacyclogenesis and parasite infectivity, opens perspectives on understanding the impact of lipid status on the development of leishmaniasis. Therefore, fatty acids and their metabolism may offer new therapeutic targets but would also be interesting for innovative nutritional interventions.

A second set of papers deals with diagnostics issues for Rocio virus and Trypanosomatidae infections.

Rocio virus infection is a neglected threat transmitted by mosquitoes. New immune inputs are required for serological diagnosis and epidemiological surveillance in low-resource settings. An in silico approach identifies a specific antigenic peptide (p_ROCV2) in the NS1 Rocio virus protein, which is predicted to be stable and exposed on the virus surface. It demonstrates the essential properties required to interact with antibodies. These findings provide insights needed to push up the development of a diagnostic platform and investigate therapeutic alternatives.

Detection and diagnosis of the human and animal infections caused by Trypanosomatidae family parasites remain challenging [19,20,21]. The isothermal amplification of nucleic acids has the potential to be applied in resource-limited areas for the detection of infectious agents, as it does not require complex nucleic purification steps or specific and expensive equipment and reagents to perform the reaction and read the results. Since human and animal infections by pathogens of the Trypanosomatidae family occur mainly in resource-limited areas with scant health infrastructures and personnel, in conjunction with non-invasive biological biopsy [8], these methodologies would therefore hold great promise. Sereno et al. [22] propose a critical literature review on applying isothermal nucleic acid amplification to detect *Trypanosoma* and *Leishmania* infections. The authors highlight gaps and propose ways to fill them to translate these powerful technologies into real-world field applications for a neglected human and animal diseases caused by Trypanosomatidae.

In the northern hemisphere, the ectoparasite *Ixodes ricinus* is a vector for tick-borne diseases (TBD) such as rickettsiosis, Lyme borreliosis, human granulocytic anaplasmosis, or the tick-borne encephalitis virus [23,24]. Owing to climate change, a temperature rise is anticipated following in the coming years. That would increase tick activity and population, spreading TBD. Consequently, it is critical to understand how microbial tick communities contribute to TBD’s fitness and occurrence. Using 16S rRNA gene amplicon sequencing, Weisinger et al. [25] analyzed bacterial taxon diversity in various tick organs and tissues (midgut, salivary glands) and the residual tick material on whole *Ixodes ricinus*. They use a DNA extraction protocol, newly developed for tick samples, and a self-designed mock community. This new approach dissecting ticks and isolating tissue—extracting tick DNA using an automated DNA extraction method and employing a tick-specific mock community—allows for the effective identification of tick-specific pathogens and endosymbionts. This approach and the attendant recommendations for using a tick-specific mock community will enable the observation of pathogens’ prevalences and abundances in tick populations. It might be used to assess the danger of TBD infections in particular forest and park areas, thus allowing hazard assessment. 

Schistosomiasis, also known as snail fever or bilharzia, is a parasitic disease caused by *Schistosoma*, a trematode flatworm. The World Health Organization considers it the second most prevalent parasitic disease after malaria, affecting more than 230 million people in over 70 countries. Infection occurs via various activities ranging from agricultural, domestic, and occupational activities to recreational activities, where the freshwater snails, *Biomphalaria*, release *Schistosoma* cercariae larvae that penetrate the skin of humans when exposed to water. Au et al. [26] review the latest molecular studies on the snail *Biomphalaria*, its ecology, evolution, and immune response. It proposes genomics as a foundation by which to understand and control this disease vector and thus, schistosomiasis transmission.

This Special Issue has provided welcome opportunities to focus on some neglected vector-borne pathologies (e.g., leishmaniasis, schistosomiasis…) from the understudied and underreported Rocio virus to various tick-borne pathogens. It will help delineate new diagnostic tools and strategies, therapeutic approaches, and future drug targets. It will also emphasize the interest of genomics to question the emergence and spread of vector-borne infections.

## References

[1] Akhoundi M., Sereno D., Marteau A., Bruel C., Izri A. (2020). Who Bites Me? A Tentative Discriminative Key to Diagnose Hematophagous Ectoparasites Biting Using Clinical Manifestations. Diagnostics.

[2] Sereno D. (2022). Epidemiology of Vector-Borne Diseases 2.0. Microorganisms.

[3] Kholoud K., Sereno D., Lahouari B., El Hidan M., Souad B. (2018). Management of Leishmaniases in the Era of Climate Change in Morocco. Int. J. Environ. Res. Public Health.

[4] Londono-Renteria B., Drame P.M., Montiel J., Vasquez A.M., Tobón-Castaño A., Taylor M., Vizcaino L., Lenhart A.E. (2020). Identification and Pilot Evaluation of Salivary Peptides from Anopheles albimanus as Biomarkers for Bite Exposure and Malaria Infection in Colombia. Int. J. Mol. Sci..

[5] Giraldo-Calderón G.I., Calle-Tobón A., Rozo-López P., Colpitts T.M., Park Y., Rua-Uribe G.L., Londono-Renteria B. (2020). Transcriptome of the Aedes aegypti Mosquito in Response to Human Complement Proteins. Int. J. Mol. Sci..

[6] Zhang J., Huang Y., Li L., Dong J., Liao M., Sun M. (2020). Transcriptome Analysis Reveals the Neuro-Immune Interactions in Duck Tembusu Virus-Infected Brain. Int. J. Mol. Sci..

[7] André S., Raja Z., Humblot V., Piesse C., Foulon T., Sereno D., Oury B., Ladram A. (2020). Functional Characterization of Temporin-SHe, a New Broad-Spectrum Antibacterial and Leishmanicidal Temporin-SH Paralog from the Sahara Frog (*Pelophylax saharicus*). Int. J. Mol. Sci..

[8] Sereno D., Akhoundi M., Sayehmri K., Mirzaei A., Holzmuller P., Lejon V., Waleckx E. (2020). Noninvasive Biological Samples to Detect and Diagnose Infections due to Trypanosomatidae Parasites: A Systematic Review and Meta-Analysis. Int. J. Mol. Sci..

[9] Jublot D., Cavaillès P., Kamche S., Francisco D., Fontinha D., Prudêncio M., Guichou J.-F., Labesse G., Sereno D., Loeuillet C. (2022). A Histone Deacetylase (HDAC) Inhibitor with Pleiotropic In Vitro Anti-Toxoplasma and Anti-Plasmodium Activities Controls Acute and Chronic Toxoplasma Infection in Mice. Int. J. Mol. Sci..

[10] Loeuillet C., Touquet B., Oury B., Eddaikra N., Pons J.L., Guichou J.F., Labesse G., Sereno D. (2018). Synthesis of aminophenylhydroxamate and aminobenzylhydroxamate derivatives and in vitro screening for antiparasitic and histone deacetylase inhibitory activity. Int. J. Parasitol. Drugs Drug Resist..

[11] Loeuillet C., Touquet B., Guichou J.F., Labesse G., Sereno D. (2019). A Tiny Change Makes a Big Difference in the Anti-Parasitic Activities of an HDAC Inhibitor. Int. J. Mol. Sci..

[12] Ennes-Vidal V., Antunes D., Poláková E., Yurchenko V., Oliveira S.S.C., Faria da Mota F., Guimaraes A.C.R., Caffarena E.R., Santos A.L.S., Branquinha M.H. (2022). Differences in Charge Distribution in *Leishmania tarentolae* Leishmanolysin Result in a Reduced Enzymatic Activity. Int. J. Mol. Sci..

[13] Leroux M., Luquain-Costaz C., Lawton P., Azzouz-Maache S., Delton I. (2023). Fatty Acid Composition and Metabolism in Leishmania Parasite Species: Potential Biomarkers or Drug Targets for Leishmaniasis?. Int. J. Mol. Sci..

[14] Sereno D., Harrat Z., Eddaikra N., Denis Sereno Z.H.N.E., Sereno D., Harrat Z., Eddaikra N. (2019). Meta-analysis and discussion on challenges to translate Leishmania drug resistance phenotyping into the clinic. Acta Trop..

[15] Seblova V., Oury B., Eddaikra N., Aït-Oudhia K., Pratlong F., Gazanion E., Maia C., Volf P., Sereno D. (2014). Transmission potential of antimony-resistant leishmania field isolates. Antimicrob. Agents Chemother..

[16] Eddaikra N., Ait-Oudhia K., Kherrachi I., Oury B., Moulti-Mati F., Benikhlef R., Harrat Z., Sereno D. (2018). Antimony susceptibility of Leishmania isolates collected over a 30-year period in Algeria. PLoS Negl. Trop. Dis..

[17] Lira R., Sundar S., Makharia A., Kenney R., Gam A., Saraiva E., Sacks D. (1999). Evidence that the high incidence of treatment failures in Indian kala-azar is due to the emergence of antimony-resistant strains of Leishmania donovani. J. Infect. Dis..

[18] Domagalska M.A., Barrett M.P., Dujardin J.C. (2023). Drug resistance in Leishmania: Does it really matter?. Trends Parasitol..

[19] Desquesnes M., Gonzatti M., Sazmand A., Thévenon S., Bossard G., Boulangé A., Gimonneau G., Truc P., Herder S., Ravel S. (2022). A review on the diagnosis of animal trypanosomoses. Parasit. Vectors.

[20] Desquesnes M., Sazmand A., Gonzatti M., Boulangé A., Bossard G., Thévenon S., Gimonneau G., Truc P., Herder S., Ravel S. (2022). Diagnosis of animal trypanosomoses: Proper use of current tools and future prospects. Parasit. Vectors.

[21] Farikou O., Simo G., Njiokou F., Kamé Ngassé G.I., Achiri Fru M., Geiger A. (2023). Trypanosome Infections and Anemia in Cattle Returning from Transhumance in Tsetse-Infested Areas of Cameroon. Microorganisms.

[22] Sereno D., Oury B., Geiger A., Vela A., Karmaoui A., Desquesnes M. (2022). Isothermal Nucleic Acid Amplification to Detect Infection Caused by Parasites of the Trypanosomatidae Family: A Literature Review and Opinion on the Laboratory to Field Applicability. Int. J. Mol. Sci..

[23] Madison-Antenucci S., Kramer L.D., Gebhardt L.L., Kauffman E. (2020). Emerging Tick-Borne Diseases. Clin. Microbiol. Rev..

[24] Nilsson P.-O., Tjernberg I. (2023). Lyme Neuroborreliosis—Significant Local Variations in Incidence within a Highly Endemic Region in Sweden. Microorganisms.

[25] Wiesinger A., Wenderlein J., Ulrich S., Hiereth S., Chitimia-Dobler L., Straubinger R.K. (2023). Revealing the Tick Microbiome: Insights into Midgut and Salivary Gland Microbiota of Female Ixodes ricinus Ticks. Int. J. Mol. Sci..

[26] Au M.F.F., Williams G.A., Hui J.H.L. (2023). Status Quo and Future Perspectives of Molecular and Genomic Studies on the Genus Biomphalaria—The Intermediate Snail Host of Schistosoma mansoni. Int. J. Mol. Sci..

